# Supplementary figures and images for: The effects of caloric restriction on adipose tissue and metabolic health are sex- and age-dependent
Source: eLife. 2023 Apr 25;12:e88080. doi: 10.7554/eLife.88080 (PMC10171869; doi:10.7554/eLife.88080)

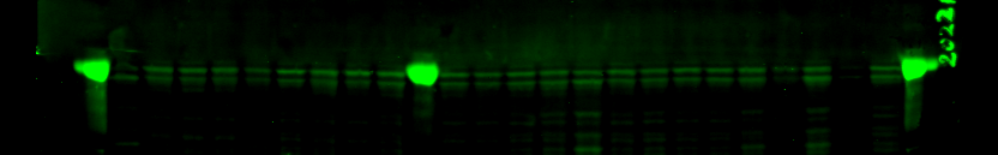

Supplement: Figure 2—source data 1. — Values for the data presented in Figure 2A–G1–J and L are shown in the file named ‘Figure 2_Source_Data.xlsx’. Raw uncropped images of immunoblots in Figure 2K are shown in the files named ‘Figure 2 blots lower beta-actin.tif’, ‘Figure 2 blots lower HSL.png’, ‘Figure 2 blots lower P-HSL.png’, ‘Figure 2 blots upper beta-actin.tif’, ‘Figure 2 blots upper HSL.png’, and ‘Figure 2 blots upper P-HSL.png’. Annotated images of uncropped immunoblots from Figure 2K are shown in the file named ‘Figure_2 K_Uncropped_blots.eps’. [file elife-88080-fig2-data1.zip › Figure 2 blots lower HSL.png]

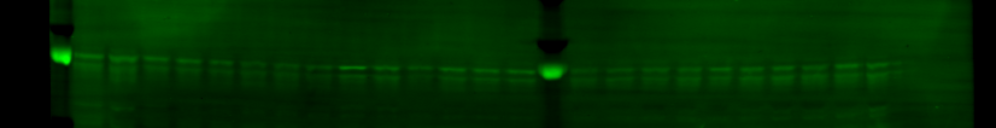

Supplement: Figure 2—source data 1. — Values for the data presented in Figure 2A–G1–J and L are shown in the file named ‘Figure 2_Source_Data.xlsx’. Raw uncropped images of immunoblots in Figure 2K are shown in the files named ‘Figure 2 blots lower beta-actin.tif’, ‘Figure 2 blots lower HSL.png’, ‘Figure 2 blots lower P-HSL.png’, ‘Figure 2 blots upper beta-actin.tif’, ‘Figure 2 blots upper HSL.png’, and ‘Figure 2 blots upper P-HSL.png’. Annotated images of uncropped immunoblots from Figure 2K are shown in the file named ‘Figure_2 K_Uncropped_blots.eps’. [file elife-88080-fig2-data1.zip › Figure 2 blots upper HSL.png]

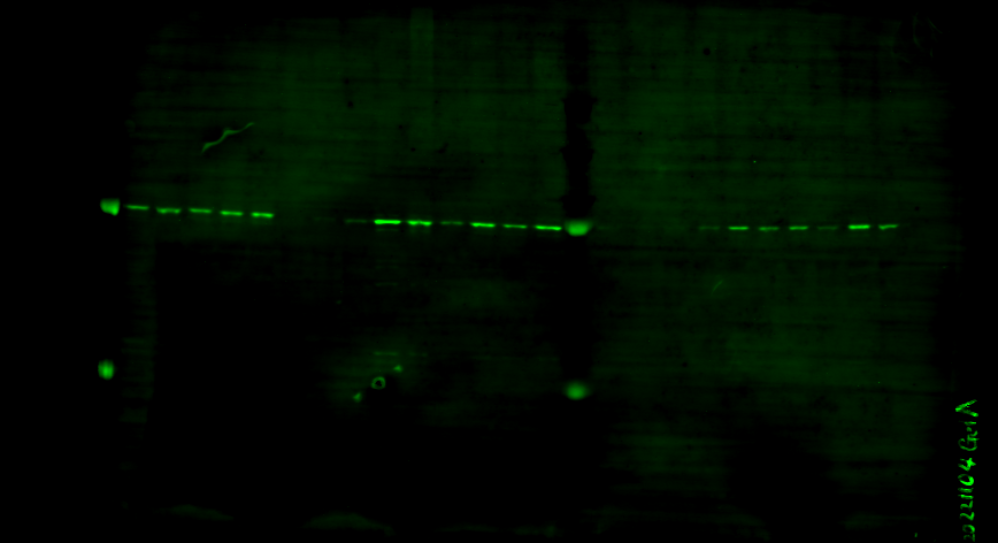

Supplement: Figure 2—source data 1. — Values for the data presented in Figure 2A–G1–J and L are shown in the file named ‘Figure 2_Source_Data.xlsx’. Raw uncropped images of immunoblots in Figure 2K are shown in the files named ‘Figure 2 blots lower beta-actin.tif’, ‘Figure 2 blots lower HSL.png’, ‘Figure 2 blots lower P-HSL.png’, ‘Figure 2 blots upper beta-actin.tif’, ‘Figure 2 blots upper HSL.png’, and ‘Figure 2 blots upper P-HSL.png’. Annotated images of uncropped immunoblots from Figure 2K are shown in the file named ‘Figure_2 K_Uncropped_blots.eps’. [file elife-88080-fig2-data1.zip › Figure 2 blots upper P-HSL.png]

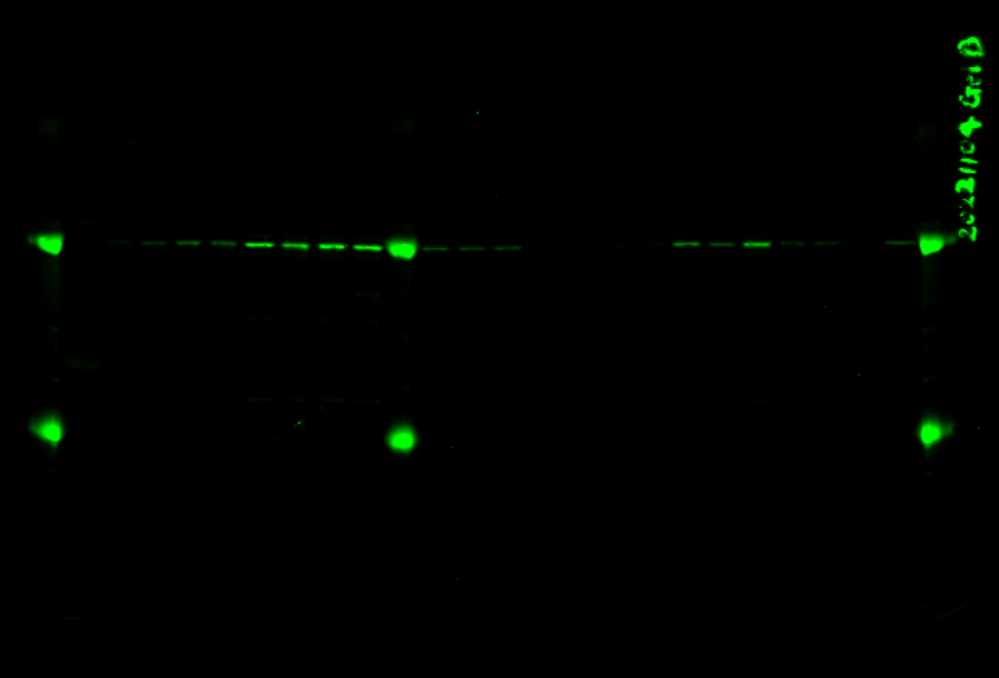

Supplement: Figure 2—source data 1. — Values for the data presented in Figure 2A–G1–J and L are shown in the file named ‘Figure 2_Source_Data.xlsx’. Raw uncropped images of immunoblots in Figure 2K are shown in the files named ‘Figure 2 blots lower beta-actin.tif’, ‘Figure 2 blots lower HSL.png’, ‘Figure 2 blots lower P-HSL.png’, ‘Figure 2 blots upper beta-actin.tif’, ‘Figure 2 blots upper HSL.png’, and ‘Figure 2 blots upper P-HSL.png’. Annotated images of uncropped immunoblots from Figure 2K are shown in the file named ‘Figure_2 K_Uncropped_blots.eps’. [file elife-88080-fig2-data1.zip › Figure 2 blots lower P-HSL.png]

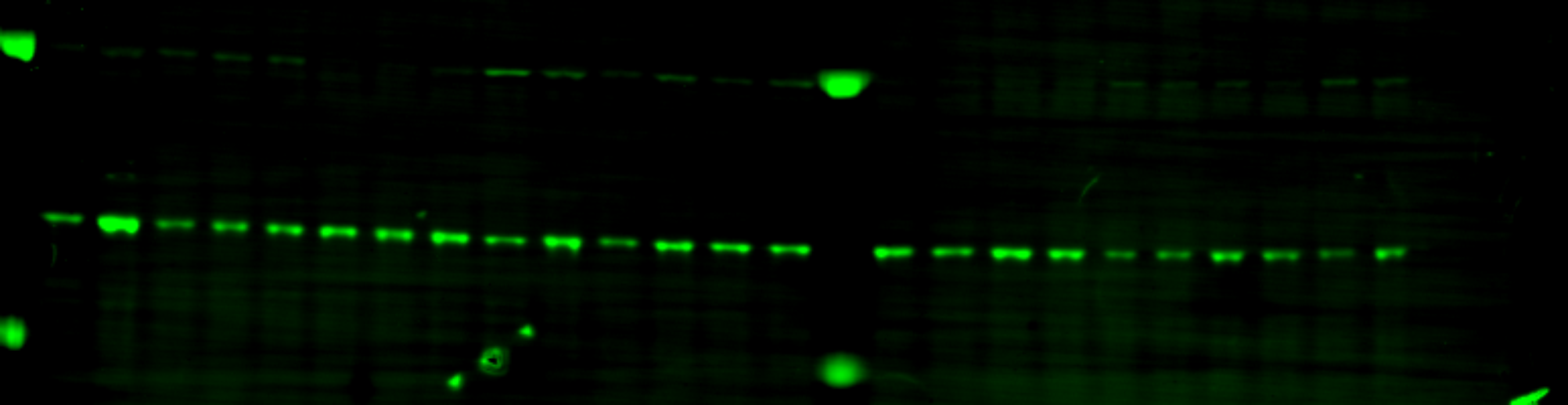

Supplement: Figure 2—source data 1. — Values for the data presented in Figure 2A–G1–J and L are shown in the file named ‘Figure 2_Source_Data.xlsx’. Raw uncropped images of immunoblots in Figure 2K are shown in the files named ‘Figure 2 blots lower beta-actin.tif’, ‘Figure 2 blots lower HSL.png’, ‘Figure 2 blots lower P-HSL.png’, ‘Figure 2 blots upper beta-actin.tif’, ‘Figure 2 blots upper HSL.png’, and ‘Figure 2 blots upper P-HSL.png’. Annotated images of uncropped immunoblots from Figure 2K are shown in the file named ‘Figure_2 K_Uncropped_blots.eps’. [file elife-88080-fig2-data1.zip › Figure 2 blots upper beta-actin.tif]

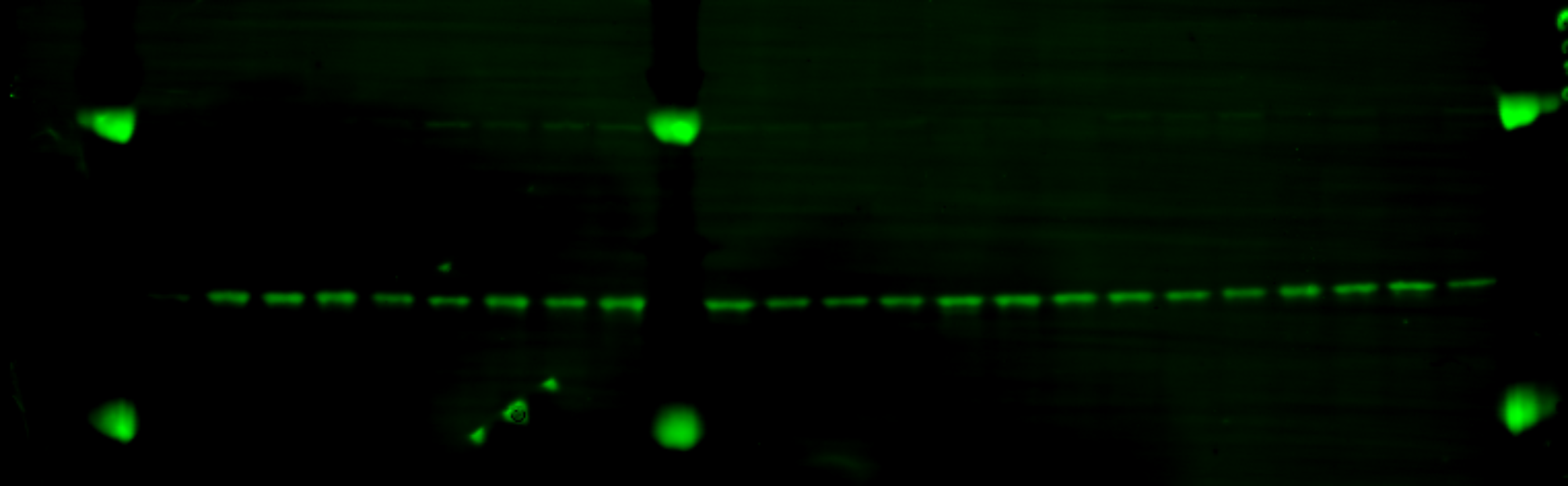

Supplement: Figure 2—source data 1. — Values for the data presented in Figure 2A–G1–J and L are shown in the file named ‘Figure 2_Source_Data.xlsx’. Raw uncropped images of immunoblots in Figure 2K are shown in the files named ‘Figure 2 blots lower beta-actin.tif’, ‘Figure 2 blots lower HSL.png’, ‘Figure 2 blots lower P-HSL.png’, ‘Figure 2 blots upper beta-actin.tif’, ‘Figure 2 blots upper HSL.png’, and ‘Figure 2 blots upper P-HSL.png’. Annotated images of uncropped immunoblots from Figure 2K are shown in the file named ‘Figure_2 K_Uncropped_blots.eps’. [file elife-88080-fig2-data1.zip › Figure 2 blots lower beta-actin.tif]
